# Luteoloside Inhibits Proliferation and Promotes Intrinsic and Extrinsic Pathway-Mediated Apoptosis Involving MAPK and mTOR Signaling Pathways in Human Cervical Cancer Cells

**DOI:** 10.3390/ijms19061664

**Published:** 2018-06-05

**Authors:** Junli Shao, Chaoxi Wang, Linqiu Li, Hairong Liang, Juanxiu Dai, Xiaoxuan Ling, Huanwen Tang

**Affiliations:** Dongguan Key Laboratory of Environmental Medicine, School of Public Health, Guangdong Medical University, Dongguan 523808, China; sjl@gdmu.edu.cn (J.S.); chaoxiwang@yeah.net (C.W.); qiuyufenglin2005@163.com (L.L.); lianghr69@163.com (H.L.); daijuanxiu@gdmu.edu.cn (J.D.); xiaoxuanling@163.com (X.L.)

**Keywords:** luteoloside, cervical cancer, proliferation, apoptosis, MAPK, mTOR

## Abstract

Cervical cancer is a common gynecological malignancy with high incidence and mortality. Drugs commonly used in chemotherapy are often accompanied by strong side-effects. To find an anti-cervical cancer drug with high effects and low toxicity, luteoloside was used to treat the cervical cancer cell line Hela to investigate its effects on cell morphology, proliferation, apoptosis, and related proteins. The study demonstrated that luteoloside could inhibit proliferation remarkably; promote apoptosis and cytochrome C release; decrease the mitochondrial membrane potential and reactive oxygen species level; upregulate the expression of Fas, Bax, p53, phospho-p38, phospho-JNK, and cleaved PARP; downregulate the expression of Bcl-2 and phospho-mTOR; activate caspase-3 and caspase-8; change the nuclear morphology, and fragmentate DNA in Hela cells. These results strongly suggest that luteoloside can significantly inhibit the proliferation and trigger apoptosis in Hela cells. In contrast, luteoloside had less proliferation inhibiting effects on the normal cell lines HUVEC12 and LO2, and minor apoptosis promoting effects on HUVEC12 cells. Furthermore, the luteoloside-induced apoptosis in Hela cells is mediated by both intrinsic and extrinsic pathways and the effects of luteoloside may be regulated by the mitogen-activated protein kinases and mTOR signaling pathways via p53.

## 1. Introduction

Cervical cancer is one of the most common gynecological malignancies, with an incidence of almost half a million new cases annually worldwide, and its global mortality is second only to breast cancer [1]. The current treatments for cervical cancer are mainly surgical resection and chemotherapy [2]. However, surgical treatment is only suitable for patients with early cervical cancer, and is ineffective for advanced cervical cancer [3]. On the other hand, drugs commonly used in chemotherapy such as cisplatin and 5-fluorouracil are expensive and have strong side-effects. Therefore, it is urgent to find new anti-cervical cancer agents with high efficiency, low toxicity, and low cost.

It has become a research hotspot to look for anti-cancer active ingredients with high curative effect and low toxicity from plants [4,5,6]. Luteoloside, a representative of natural flavonoid substance, exists in plants widely. Its content has been used as the quality control index for several Chinese herbal medicines such as Lonicera japonica Flos, Flos Chrysanthemi, and Calyx seu Fructus Physalis. Luteoloside has been reported to possess a series of biological activities such as preventing osteolysis and suppressing osteoclastogenesis [7], anti-diabetic effects [8], protective effect against doxorubicin-induced cardiotoxicity [9], antiviral activity [10], estrogenic activity [11], and antitumor potential against liver cancer [12,13] and colon cancer [14], etc. However, there has been no report about the effects of luteoloside on cervical cancer.

In the present study, we investigated the anti-cervical cancer potential of luteoloside using Hela cells as the cellular model. The Hela cell line, a very important tool in medical research field, was isolated from cervical cancer tissue taken from an American black woman with the name Henrietta Lacks. Robust growth is one of the typical characteristics of the Hela cell line [15]. Thus, the effects of luteoloside on the proliferation and apoptosis of Hela cells and the underlying mechanisms were explored here.

## 2. Results

### 2.1. Luteoloside Inhibits Proliferation and Alters Cell Morphology of Hela Cells

**Figure 1:**
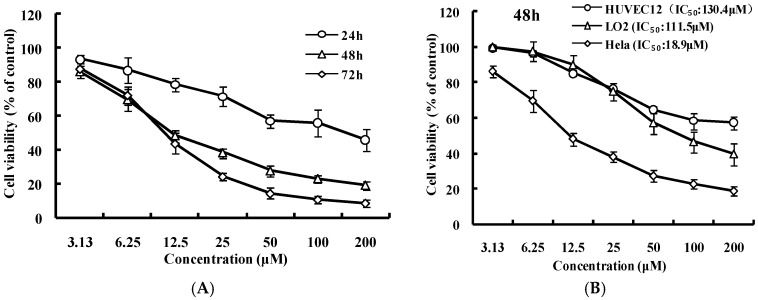

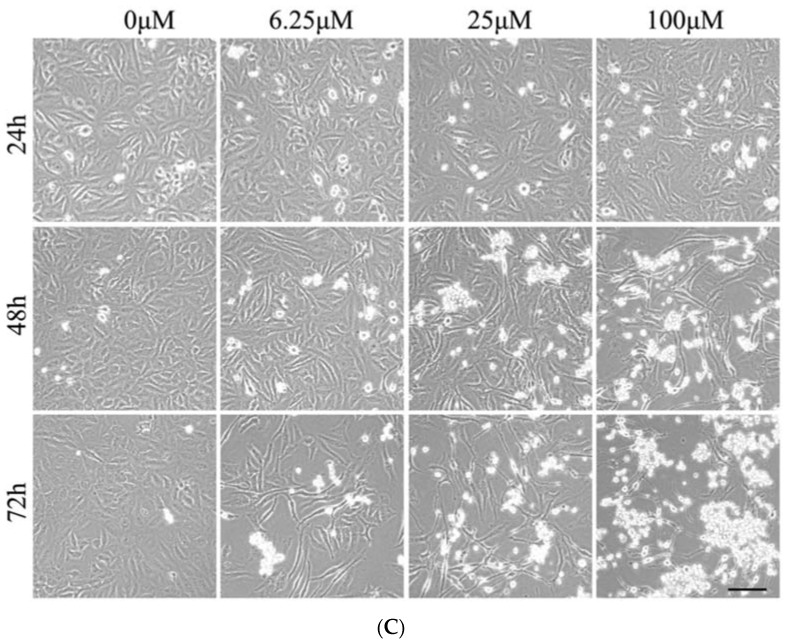
Effects of luteoloside on cell proliferation and cell morphology. Cells (**A**: Hela. **B**: HUVEC12, LO2, and Hela) were treated with luteoloside at 3.13, 6.25, 12.5, 25, 50, 100, and 200 μM for 24 (**A**), 48 (**A**,**B**) and 72 h (**A**). Cell growth was determined using cell counting kit-8. Data are the mean ± SD of three independent experiments. (**C**) Hela cells were treated with luteoloside at 0, 6.25, 25, and 100 μM for 24, 48, and 72 h and photographed. Bar = 25 μm.

To investigate the anti-cervical cancer potential of luteoloside, the anti-proliferative effect of luteoloside on Hela cells was evaluated after treatment with luteoloside. As shown in Figure 1A, luteoloside inhibited cell growth on Hela cells in a dose- and time-dependent manner. In contrast, luteoloside had less effects on the normal cell lines HUVEC12 and LO2 than on the Hela cells when treated for 48 h at the tested concentrations (Figure 1B), with the half maximal inhibitory concentration (IC_50_) values of 130.4, 111.5, and 18.9 μM for HUVEC12, LO2, and Hela cells, respectively.

Additionally, the morphology of living Hela cells (gray and slender) became increasingly slender and the proportion of dead Hela cells (white and punctate) increased gradually along with the increase of treatment concentration and time (Figure 1C).

### 2.2. Luteoloside Induces Apoptosis of Hela Cells

**Figure 2:**
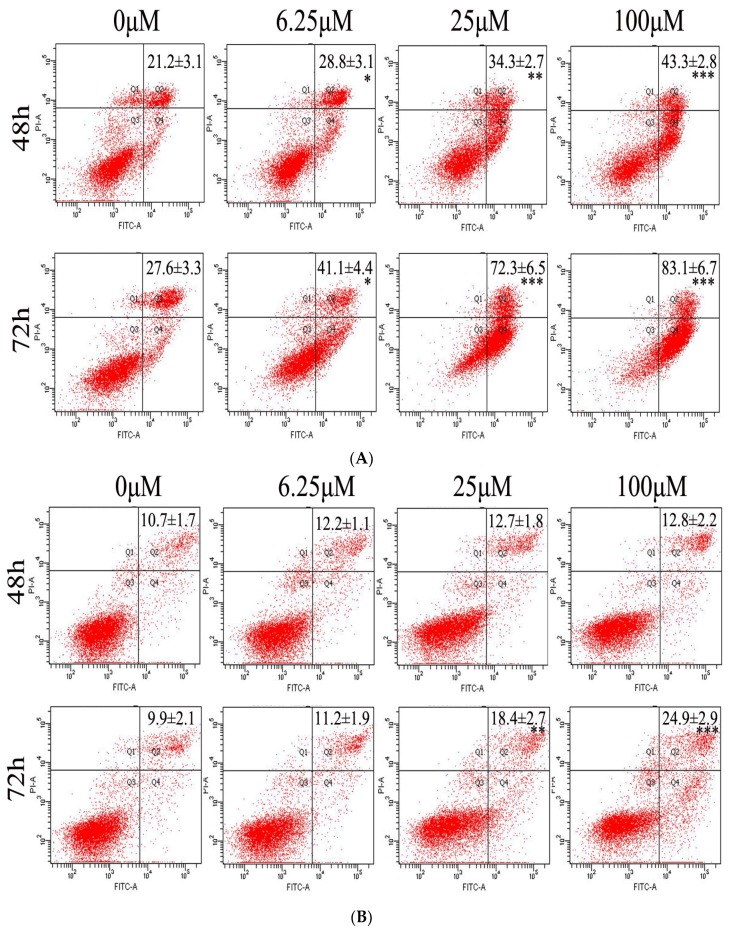
Effects of luteoloside on cell apoptosis. Hela (**A**) and HUVEC12 (**B**) cells were treated with 0, 6.25, 25, and 100 μM luteoloside for 48 or 72 h. The cells were then harvested and stained with annexinV-fluorescein isothiocyanate (FITC) and propidium iodide (PI), followed by flow cytometric analysis. The data are the percentages of apoptosis cells (upper plus lower right quadrants), expressed as the mean ± SD of three independent experiments. * *p* < 0.05, ** *p* < 0.01 and *** *p* < 0.001, versus the control group (0 μM luteoloside).

Externalization of phosphatidylserine on the cell membrane is considered to be a hallmark of the early to middle stages of apoptosis [16]. To further determine whether luteoloside induced apoptosis in Hela cells, the membrane alterations were analyzed using flow cytometry after annexinV-fluorescein isothiocyanate (FITC) and propidium iodide (PI) staining. The results demonstrated that luteoloside significantly increased the percentages of Hela cells undergoing apoptosis in a concentration- and time-dependent manner (*p* < 0.05, 0.01, or 0.001) (Figure 2A). Interestingly, no significant increase in apoptosis was observed when the normal cell line HUVEC12 was treated with luteoloside at the indicated concentrations and incubation time (*p* > 0.05), except at 25 (*p* < 0.01) and 100 μM (*p* < 0.001) for 72 h treatment (Figure 2B). Therefore, it was suggested that the apoptosis-inducing effect of luteoloside was specific to Hela cells.

### 2.3. Luteoloside Induces Apoptosis of Hela Cells through Mitochondria Pathway 

**Figure 3:**
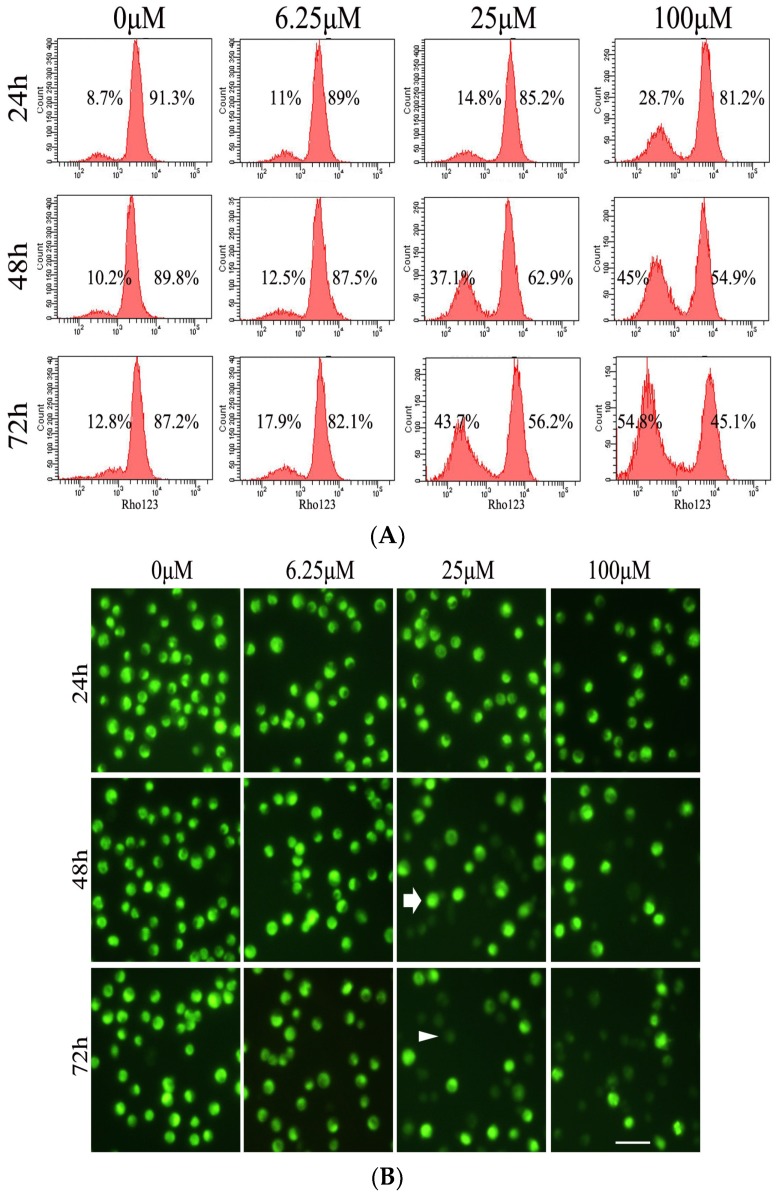
Effects of luteoloside on the mitochondria of Hela cells. (**A**) Hela cells were treated with 0, 6.25, 25, and 100 μM luteoloside for 24, 48, or 72 h, and then harvested and stained with Rhodamine 123, followed by flow cytometric analysis. The data left and right are the percentages of the cells with low and high fluorescence intensity respectively; (**B**) The cells were treated as described in (**A**) and observed under a fluorescence microscope. The arrow and arrowhead indicate the cells with high and low fluorescence intensity respectively. Bar = 25 μm.

To further investigate whether the dysfunction of mitochondria occurred in the luteoloside-induced apoptosis, the mitochondrial membrane potential (MMP) was analyzed with flow cytometry and observed under a fluorescence microscope after Rhodamine 123 staining. As shown in Figure 3A, the percentages of the cells with low (high) fluorescence intensity gradually increased (decreased) along with the treatment concentration and time increase. The total fluorescence intensity of the cells treated with luteoloside also gradually weakened in a dose- and time-dependent manner (Figure 3B). These results indicated that luteoloside treatment enhanced the permeability of the mitochondria membrane and caused the dissipation of MMP in Hela cells.

**Figure 4:**
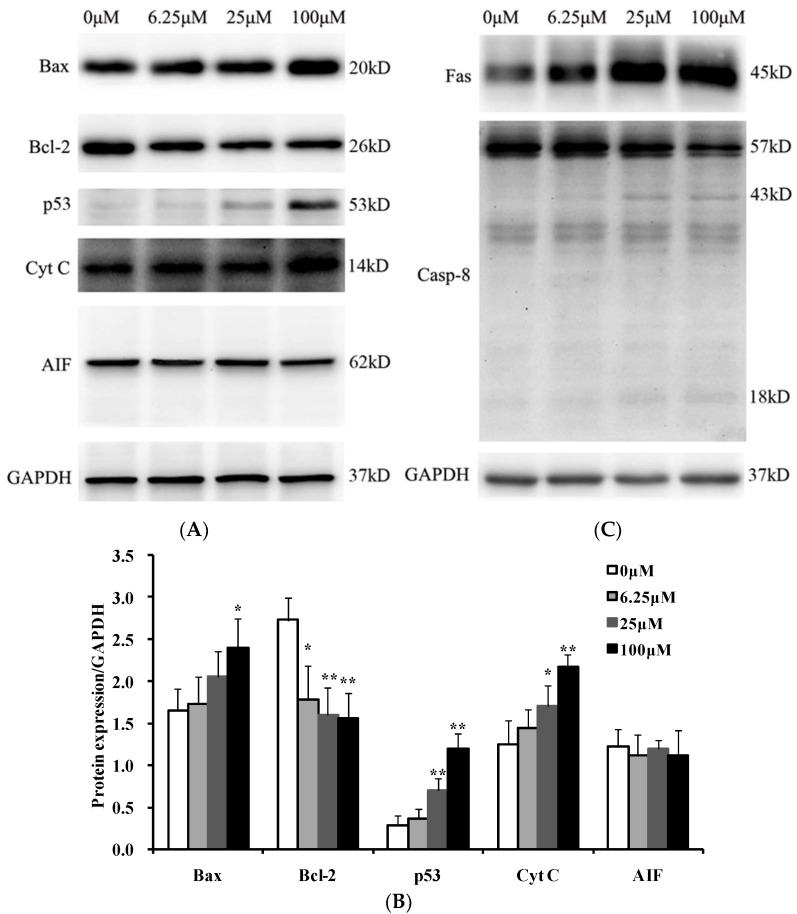

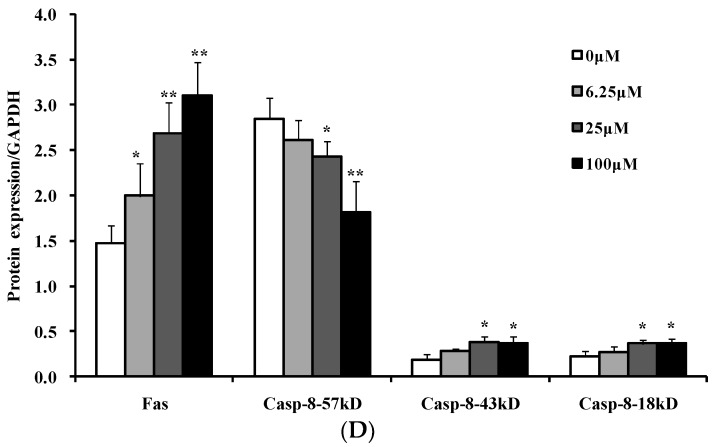
Effects of luteoloside on the apoptosis-related proteins of Hela cells. (**A**,**C**) Protein samples of the Hela cells treated with 0, 6.25, 25, and 100 μM luteoloside for 24 h were subjected to Western blot analysis. Glyceraldehyde-3-phosphate dehydrogenase (GAPDH) served as the internal control. Shown are representative results of three independent experiments. (**B**,**D**) The relative expression of proteins compared with GAPDH. Cyt C: cytochrome C. AIF: apoptosis-inducing factor. Casp-8: Caspase-8. * *p* < 0.05, ** *p* < 0.01, versus the control group (0 μM luteoloside).

Since the permeability of mitochondrial membrane was enhanced (Figure 3), the expression level of Bax and Bcl-2, two members of Bcl-2 family proteins residing in the outer mitochondrial membrane, was determined by Western blot analysis. As shown in Figure 4A,B, the expression of Bax was upregulated and the expression of Bcl-2 was suppressed in a dose-dependent manner when the cells were treated with luteoloside for 24 h. Accordingly, the p53 protein, a direct transcription activator of Bax gene [17,18] and a special inhibitor for Bcl-2 expression [19,20], was also dramatically increased dose-dependently when Hela cells were exposed to luteoloside for 24 h.

The enhancing of mitochondrial membrane permeability can cause the consequent release of cytochrome C from the mitochondria to the cytoplasm. As expected, cytochrome C in the cytoplasm increased obviously when cells were treated with luteoloside for 24 h (Figure 4A,B). Release of apoptosis-inducing factor (AIF) from the mitochondria is a hallmark event of the caspase-independent apoptosis, downstream of the mitochondrial [21]. However, the level of AIF in the mitochondria (62 kDa) did not exhibit obvious change and AIF in cytosol (57 kDa) was not detected when the Hela cells were treated with luteoloside for 24 h (Figure 4A,B). For the expression of AIF, the same results were obtained after treatment for 48 h and 72 h, which indicated that no translocation of AIF occurred. Therefore, it can be inferred that luteoloside-induced apoptosis in Hela cells is not via the caspase-independent pathway.

### 2.4. Luteoloside Induces Apoptosis of Hela Cells through the Death Receptor Pathway

To investigate whether the death receptor pathway also participated in the luteoloside-induced apoptosis, protein levels of factor associated suicide (Fas) and caspase-8 were assayed. As shown in Figure 4C,D, the Fas protein was significantly unregulated in a dose-dependent manner after luteoloside treatment for 24 h. Accordingly, the full length (57 kDa) of caspase-8 decreased and the 43 (18) kDa fragments of caspase-8 increased in a dose-dependent manner. Therefore, the death receptor pathway was also involved in the luteoloside-induced apoptosis.

### 2.5. Luteoloside Activates Caspase-3

**Figure 5:**
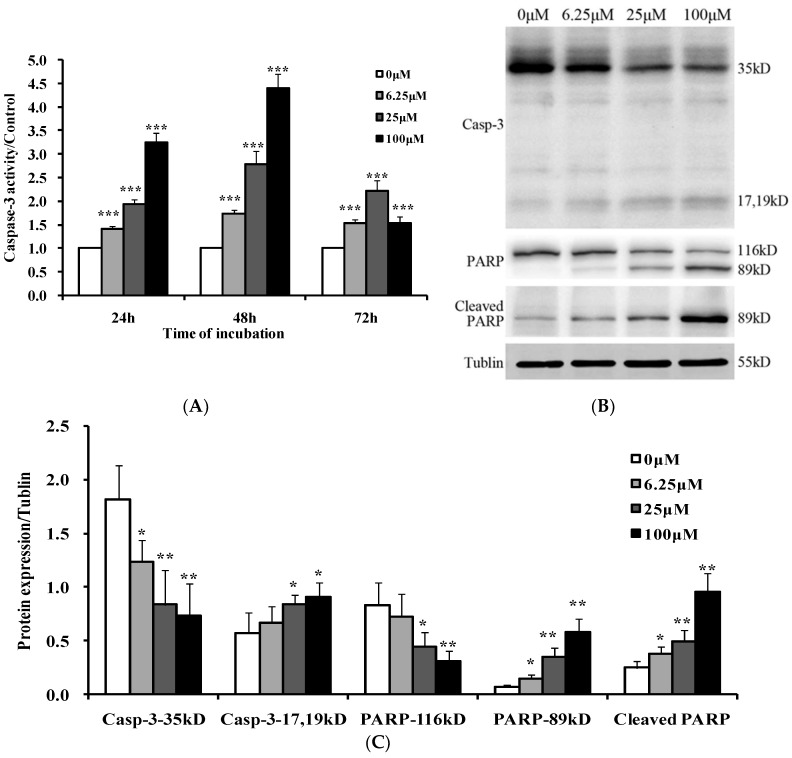
Effects of luteoloside on the activity of caspase-3 and the cleavage of poly (ADP-ribose) polymerase (PARP). (**A**) Cells were treated with various concentrations of luteoloside for the indicated time. The activity of caspase-3 was then determined and expressed as the fold over the control (0 μM luteoloside); (**B**) Cells were treated with the indicated concentrations of luteoloside for 24 h. The cleavage of caspase-3 and PARP were analyzed by Western blot using the specific antibodies. The level of tublin was used as an internal control. Results shown are representative of three independent experiments; (**C**) The relative expression of proteins compared with tublin. Casp-3: Caspase-3. The data are the mean ± SD of three independent experiments. * *p* < 0.05, ** *p* < 0.01, and *** *p* < 0.001 represent statistically significant differences versus control group (0 μM luteoloside).

Since caspase-3 is known as an executioner caspase in apoptosis and its activation is a crucial event leading to apoptosis [22], the activity of caspase-3 in Hela cells treated with luteoloside was measured. It was found that luteoloside induced the activation of caspase-3 in a concentration- and time-dependent manner at the tested concentration for 24 and 48 h treatments (Figure 5A). The activity of caspase-3 of Hela cells treated with luteoloside for 72 h was higher than that of control, but lower than that of the 24 or 48 h, which should be due to the enzyme deactivation at late apoptosis.

It is known for caspases-3 that the activation event is proteolytic cleavage. As expected, the full length (35 kDa) of caspase-3 decreased and the cleaved 17(19) kDa fragments increased in a dose-dependent manner after luteoloside treatment for 24 h (Figure 5B,C).

Poly (ADP-ribose) polymerase (PARP), a DNA repair enzyme, will be cleaved by caspase-3 to inactivate it in cells undergoing apoptosis [23]. Thus, whether PARP was cleaved in luteoloside-treated Hela cells was determined by Western blot analysis. As shown in Figure 5B, PARP was cleaved from 116 to 89 kDa after luteoloside treatment for 24 h. Moreover, the cleaved fragments increased as the treating concentration went up, which was evidenced by two specific antibodies. One antibody could recognize not only the full length PARP but also the cleaved fragments. The other could recognize only the cleaved fragments.

### 2.6. Luteoloside Changes the Nuclear Morphology and Fragmentates DNA of Hela Cells 

**Figure 6:**
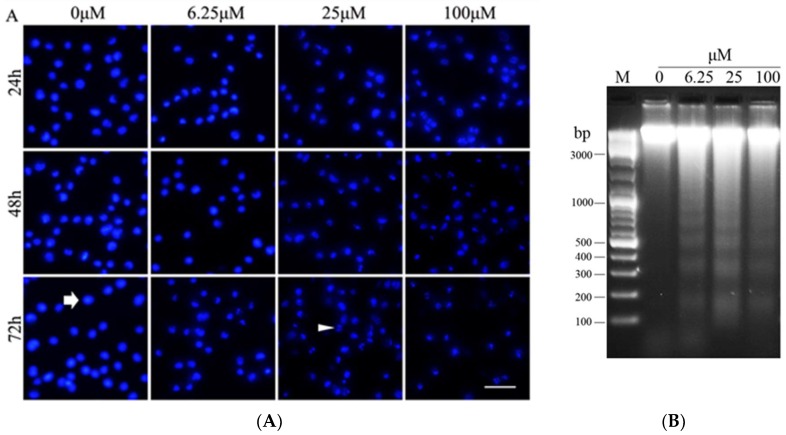
Effects of luteoloside on nuclear morphology and DNA fragmentation in Hela cells. (**A**) The cells treated with or without luteoloside were stained with Hoechst 33342 and observed under a fluorescence microscope. The arrow indicates the cells with round and intact nuclei. The arrowhead indicates the cells with the typical characteristics of apoptosis. Bar = 25 μm; (**B**) Total genomic DNA was isolated from Hela cells treated with or without luteoloside for 48 h. DNA fragmentation was analyzed by agarose gel electrophoresis.

To examine the alteration of nuclear morphology in response to luteoloside treatment, Hela cells treated with or without luteoloside were stained with the DNA dye Hoechst 33342 and visualized by fluorescent microscopy. As shown in Figure 6A, most of the untreated cells showed round and intact nuclei. In contrast, the proportion of the cells with the typical characteristics of apoptosis including bright staining and condensed chromatin and punctate apoptotic bodies rose gradually as the treatment concentration and time increased. Accordingly, the typical DNA ladder patterns for apoptosis were observed in total genomic DNA samples of Hela cells treated by luteoloside for 48 h (Figure 6B).

### 2.7. Luteoloside Decreases the Intracellular Reactive Oxygen Species (ROS) of Hela Cells

**Figure 7:**
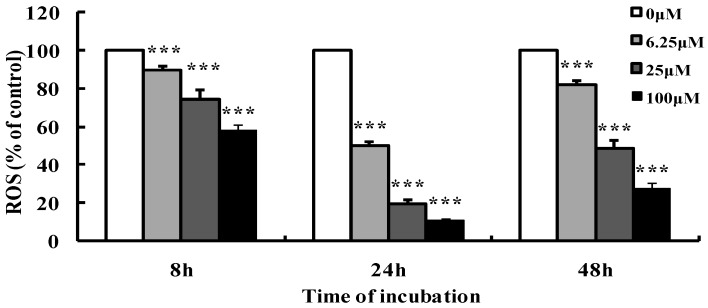
Effects of luteoloside on intracellular reactive oxygen species (ROS) levels in Hela cells. Cells were treated with various concentrations of luteoloside for the indicated time, and then incubated with 10 μM 2′,7′-dichlorodihydrofluorescein diacetate for 20 min. 2′,7′-dichlorodihydrofluorescein fluorescence was assayed on a microplate reader. ROS levels were given as the percentage of control without luteoloside treatment. The data are the mean ± SD of three independent experiments. *** *p* < 0.001 versus the control group.

ROS have always been considered closely related with cancer [24] and are involved in the anti-tumor activities of some compounds. To investigate whether ROS participated in the luteoloside’s anti-cancer activity on Hela cells, the intracellular ROS levels were assessed in Hela cells treated with luteoloside. As a result, luteoloside could markedly decrease the ROS levels of Hela cells in a dose and time-dependent manner for 8 and 24 h treatment (Figure 7). ROS levels of Hela cells treated with luteoloside for 48 h were also decreased in a dose-dependent manner, but they were higher than that of 24 h, which should be due to the acceleration of ROS generation at late apoptosis.

### 2.8. Luteoloside Regulates the Mitogen-Activated Protein Kinases (MAPKs) and Mammalian Target of Rapamycin (mTOR) Signaling Pathways

**Figure 8:**
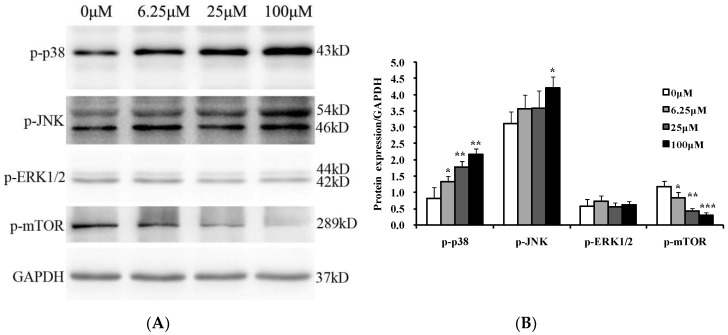
Effects of luteoloside on the proteins of mitogen-activated protein kinases and mammalian target of rapamycin signal pathways. (**A**) Hela cells were treated with 0, 6.25, 25, and 100 μM luteoloside for 24 h. Protein samples were extracted and subjected to Western blot analysis with the specific antibodies. Glyceraldehyde-3-phosphate dehydrogenase (GAPDH) served as internal control; (**B**) The relative expression of proteins compared with GAPDH. Shown are representative results of three independent experiments. The quantity of p-JNK includes the 46 kD and 54 kD two bands. The quantity of p-ERK1/2 includes the 42 kD and 44 kD two bands. p-: phospho-. JNK: c-Jun N-terminal kinase. ERK1/2: external-signal regulated kinase1/2. mTOR: mammalian target of rapamycin. * *p* < 0.05, ** *p* < 0.01, and *** *p* < 0.001, versus the control group (0 μM luteoloside).

MAPKs can respond to a variety of extracellular stimuli and mediate many kinds of cell behaviors. The p38, c-Jun N-terminal kinase (JNK), and external-signal regulated kinase1/2 (ERK1/2), three main subgroups among the MAPKs family, function as a nexus of cellular signal transduction cascades and play an important role in cell growth and apoptosis [25]. The mTOR is an important regulatory factor of the cellular signaling network and can be activated by several factors. It is involved in cell growth, proliferation, apoptosis, and many other biological processes mainly through phosphoinositide 3-kinase (PI3K)/Akt/mTOR signaling pathway [26]. Therefore, the phosphorylation of MAPKs and mTOR were analyzed by Western blot analysis. As shown in Figure 8, luteoloside treatment for 24 h resulted in a dose-dependent increase in the levels of phospho-p38 and phospho-JNK, while there was little effect on the level of phospho-ERK1/2. Similar results were obtained for the phospho-ERK1/2 level after 48 and 72 h exposure to luteoloside. In contrast to the levels of phospho-p38 and phospho-JNK, the phosphorylation level of mTOR was significantly decreased after the Hela cells were exposed to luteoloside for 24 h. These results suggest that the MAPK and mTOR signaling pathways are involved in the effects of luteoloside.

## 3. Discussion

Normally, the proliferation and apoptosis of cells maintain a dynamic balance and can lead to oncogenesis when proliferation accelerates and apoptosis decreases. Therefore, inhibiting proliferation and promoting apoptosis are important mechanisms of many antitumor agents [27,28]. The present study demonstrated that luteoloside had a good performance in inhibiting proliferation and promoting apoptosis of Hela cells. Furthermore, the luteoloside-induced apoptosis involved two main apoptosis pathways, and the effects of luteiloside were related to the MAPK and mTOR signaling pathways.

In addition to the anticarcinogenic effect, the magnitude of the side-effect is also an important factor in the evaluation of an anti-cancer agent. This study indicates that luteoloside can inhibit the proliferation of Hela cells effectively in a dose- and time-dependent manner (Figure 1A) with an IC_50_ 18.9 μM for 48 h treatment. On the other hand, luteoloside has a lesser effect on the growth of the normal cell HUVEC12 and LO2 at the tested concentrations (Figure 1B) with the IC_50_ 130.4 μM and 111.5 μM for 48 h treatment, respectively. In contrast, proliferation inhibition of luteoloside on several hepatocellular carcinoma cells [12], chronic myeloid leukemia cell K562 [29], colon carcinoma cell COLO 320 DM, and normal cell VERO [14] are also dose- and time-dependent with a IC_50_ of approximately 100, 200, 266, and 854 μM, respectively, for 48 h treatment. Therefore, luteoloside may be a promising candidate for anti-cervical cancer agents with the advantages of high efficiency and low toxicity.

Apoptosis occurs through the following two main classic apoptotic pathways [30,31]. One, the extrinsic pathway, i.e., death receptor pathway: under the stimulation of external factors, the death ligand (e.g., Fas ligand) and the death receptor (e.g., Fas) bind together, followed by the coupling of the death receptors with caspase-8 by an adapter protein. Thus, apoptosis will be initiated after the subsequent activation of caspase-8 [32]. The other, the intrinsic pathway, i.e., mitochondrial pathway: under the pressure signal such as DNA damage the ratio of pro-apoptotic protein and anti-apoptotic protein of Bcl-2 family proteins in outer mitochondrial membrane will increased, which will lead to the loss of MMP and opening of the permeability transition pore. Then the subsequent cytochrome C release from the mitochondria to the cytoplasm will trigger the downstream apoptotic events [33]. The present study showed that the expression of Fas increased and caspase-8 was activated by proteolytic cleavage in luteoloside-treated Hela cells (Figure 4C,D). On the other hand, an upregulated ratio of Bax/Bcl-2 (Figure 4A,B), loss of MMP (Figure 3A,B), and elevated level of cytosolic cytochrome C (Figure 4A,B) were also observed in the Hela cells exposed to luteoloside. Thus, luteoloside-induced apoptosis involved the death receptor pathway and the mitochondrial pathway.

p53 is a well-known tumor suppressor protein and there is a close relationship between p53 and cell apoptosis. One hand, p53 can promote the expression of Bax gene [17,18] and suppress the expression of Bcl-2 [19,20] specifically, i.e., enhance the ratio of Bax/Bcl-2, an important symbol of apoptosis via the mitochondrial pathway. On the other hand, the expression of the death receptor Fas, a vital molecular of death receptor pathway, is dependent on p53 [34]. As expected, luteoloside not only induced cell apoptosis but also upregulated p53 expression (Figure 4A,B) in Hela cells. In addition, luteoloside-induced DNA damage (Figure 6) might increase the p53 level in turn as DNA damage is a known p53 inducer [35].

The caspase family plays an important role in the process of cell apoptosis where caspase-3 is best known as a critical executioner of apoptosis. After activated by caspase-8 or caspase-9, caspase-3 is partially or totally responsible for the proteolytic cleavage of many key proteins such as the nuclear enzyme PARP [23]. In the late stages of apoptosis, the typical morphological signs will occur including chromatin condensation, cell shrinkage, nuclear and DNA fragmentation and, finally, membrane blebbing to form apoptotic bodies [36]. In this study, caspase-3 was activated by luteoloside as confirmed by activity assay (Figure 5A) and Western blot analysis for the cleavage of caspase-3 and PARP (Figure 5B,C). Furthermore, the main typical characteristics of apoptosis were also demonstrated by Hoechst 33342 staining (Figure 6A) and DNA ladder pattern analysis (Figure 6B).

The death receptor pathway and mitochondrial pathway above-mentioned are all caspase-dependent pathways. In addition, there is a AIF-involved apoptosis pathway independent of caspase [37]. In response to proapoptotic stimuli, AIF will be cleaved from 62 to 57 kDa, released from the mitochondrial intermembrane space to the cytoplasm, subsequently translocated to the nucleus and finally evoke caspase-independent apoptosis [38]. In the present study, we showed that luteoloside had no visible effect on AIF levels in both mitochondria (62 kDa) and cytosol (57 kDa) (Figure 4A,B), which might be because the apoptotic activity of AIF is cell type and stimuli-dependent.

Intracellular ROS are constantly generated and eliminated during the cell’s metabolic processes and there is a delicate balance between generation and elimination under normal physiological conditions. After the balance is broken, elevated ROS levels will promote the occurrence of malignant tumor [24]. Recent studies have suggested that luteoloside’s effects on intracellular ROS in different cancer cells are not necessarily the same, significantly increasing [13] or reducing [12,14], though luteoloside exhibited anti-tumor activity in both cases. For the second case, Fan et al. had shown that the luteoloside-induced decrease of ROS inhibited the expression of the nucleotide-binding domain, leucine-rich family (NLR), pyrin-containing 3 (NLRP3) inflammasome, subsequent caspase-1 activation and interleukin-1beta secretion, which resulted in the suppression of proliferation and metastasis of Hepatocellular carcinoma [12]. NLRP3 inflammasome activation plays an important role in the proliferation, survival, migration or invasiveness of many cancer cells [39,40,41]. ROS can activate the NLRP3 inflammasome, and inhibitors or scavengers of ROS can inhibit NLRP3 inflammasome activation [42,43]. In view of these previous studies, it was speculated that the luteoloside-induced decrease of ROS levels (Figure 7) in this study might contribute to the anti-proliferative effect on Hela cells via NLRP3 inflammasome inhibition.

The MAPK signaling pathway family are vital intracellular signaling system. They response to multiple extracellular signals or stimuli and regulate a variety of important physiological processes including proliferation, differentiation, apoptosis or survival, inflammation, and stress responses, etc. [25]. Among the MAPK signaling pathways, the ERK1/2 pathway, the JNK pathway, and the p38 MAPK pathway are three most important and well-characterized pathways [25,44]. Numerous studies have shown that activation of ERK1/2 pathway could enhance cell proliferation and survival [45], while the activation of the JNK and p38 pathways mainly promotes apoptosis. Many potential anticancer agents [46,47] and some common drugs [48,49] for cancer treatment can activate JNK and p38 MAPK pathways in the meanwhile, and then induce apoptosis of cancer cells. In the present study, we showed that luteoloside could also activate both JNK and p38 pathways as evidenced by the enhanced phosphorylation levels, whereas the phosphorylation levels of ERK1/2 did not show obvious change (Figure 8).

It has been proved that JNK can activate p53 by phosphorylation [50]. Another MAPK p38 was demonstrated to associates physically with p53 and its activation can stimulate the transcriptional activity of p53 [51,52]. Therefore, luteoloside-induced apoptosis was most probably regulated by JNK and p38, and the regulation was likely mediated by p53.

mTOR, an important regulator of the intracellular signaling network, is involved in cell growth, proliferation, apoptosis, migration, autophagy, and many other biological processes mainly through PI3K/Akt/mTOR pathway [53]. Activation of the PI3K/Akt/mTOR signaling pathway could inhibit apoptosis induced by various stimuli and promote cell proliferation, and thus plays an important role in the initiation, development, and drug resistance of malignant tumors [54,55]. Therefore, it has become the focus of research in tumor therapy to inhibit cell proliferation or promote apoptosis through inhibiting PI3K, Akt, mTOR, and related genes by gene knockout or small molecule drug intervention [26,56,57]. In cervical cancer, the PI3K/Akt/mTOR pathway is often dysregulated and is considered a promising target for therapy [58]. Interestingly, the phospho-mTOR level could be downregulated remarkably by luteoloside in this study. Therefore, luteoloside might be a promising candidate agent against cervical cancer targeting for mTOR. Regarding the mechanism, the study of Jung et al. indicated that it is through increasing the expression of Bax, cleaved-caspase-3, and cleaved-PARP as well as decreasing the expression of Bcl-2 for the inhibition of the PI3K/Akt/mTOR pathway to promote apoptosis [59].

The present study showed that JNK, p38, mTOR, and p53 were involved in the proliferation inhibition and apoptosis promotion effects of luteoloside on Hela cells, while the more explicit relationship among them and the anti-cervical cancer effects of luteoloside in vivo should be further studied.

## 4. Material and Methods

### 4.1. Cell Culture and Reagents

The Henrietta Lacks strain of cancer cells Hela was kindly provided by Cell Bank, Chinese Academy of Sciences (Shanghai, China). Human normal cells HUVEC12 (human umbilical vein endothelial cell) and LO2 (hepatocyte) were obtained from the American Type Culture Collection (Manassas, VA, USA). Three kinds of cells were all cultured in Dulbecco’s Modified Eagle Media (DMEM) supplemented with 10% fetal bovine serum at 37 °C in a humidified 5% CO_2_ atmosphere. Antibodies against caspase-3, cytochrome C, phospo-JNK, phospho-p38 MAPK, phospho-mTOR, phospho-ERK1/2, cyclinB1, Bax, and Bcl-2were purchased from Cell Signaling (Boston, MA, USA). Antibodies against p53 and PARP were purchased from Santa Cruz Biotechnology (Santa Cruz, CA, USA). Glyceraldehyde-3-phosphate dehydrogenase (GAPDH) antibody, horseradish peroxidase-conjugated secondary antibody, DNA ladder extraction kit, Gel-Red and fluorescence probe 2′,7′-dichlorodihydrofluorescein diacetate (DCFH-DA) were purchased from Beyotime Biotechnology (Beijing, China). Cell counting kit-8 (CCK-8) and annexin V-FITC apoptosis detection kit were purchased from DoJindo (Kumamoto, Japan). Rhodamine 123 and Hoechst 33342 were purchased from Sigma-Aldrich (St. Louis, MI, USA). The caspase-3 assay kit was purchased from Abcam (Cambridge, Britain). Radio immunoprecipitation assay (RIPA) lysis buffer and the bicinchoninic acid (BCA) protein assay kit were purchased from CWBIO Biotechnology (Beijing, China). Luteoloside was purchased from Shanghai Winherb Medical Technology Co., Ltd. (Shanghai, China). Luteoloside was dissolved in dimethyl sulfoxide (DMSO) at 3.13, 6.25, 12.5, 25, 50, 100, 200 mM as stock solutions and stored at −20 °C and diluted 1000 times with media as work solution when used.

### 4.2. Cell Viability Assay

Cells were plated in 96-well plates at a density of 3 × 10^3^ cells per well. Following overnight adherence, the media were exchanged with fresh media containing various doses of luteoloside. Cells incubated in media containing 0.1% DMSO were used as controls. After treatment for 24, 48, and 72 h, cell viability was assessed using a CCK-8. In brief, the culture solution in each well was exchanged with a mixture of 100 μL fresh media and 10 μL CCK-8 reagent. After incubation for another 2 h at 37 °C, absorbance was then measured on a microplate reader (BioTek Synergy2, BioTek Instruments Inc., Winooski, VT, USA) at 450 nm. The assays were all done in triplicate at least and repeated three times.

### 4.3. Annexin V-FITC/PI Double Staining

The percentage of apoptosis cells was measured by detecting the surface exposure of phosphatidylserine in apoptotic cells using an AnnexinV-FITC apoptosis detection kit according to the manufacturer’s instructions. Briefly, cells in 2.5 mL media were seeded in 6-well plates. Following overnight adherence, the media were exchanged with fresh media containing various doses of luteoloside (0, 6.25, 25, and 100 μM). After incubation for 48 or 72 h, the floating and adherent cells were harvested, washed twice in phosphate buffer saline (PBS), and re-suspended in binding buffer. AnnexinV-FITC and PI were then added into the samples. After incubation for 15 min at room temperature in the dark, the samples were diluted with binding buffer again and the percentage of apoptotic cells was analyzed within 1 h by flow cytometry. The annexin V+/PI- (the early apoptosis) and the annexin V+/PI+ (the late apoptosis) apoptotic cell populations were measured. All experiments were measured in duplicate and repeated three times.

### 4.4. Rhodamine 123 Staining

MMP-sensitive cationic dye Rhodamine 123 was used to analyze the MMP. In brief, cells treated with luteoloside as described in Section 4.3 including floating and adherent cells were collected, washed with PBS, incubated with Rhodamine 123 (10 μg/mL) in DMEM media for 30 min at 37 °C. The cells were then washed again with PBS three times, and the fluorescence intensity was detected using flow cytometry and observed and photographed under a fluorescence microscope.

### 4.5. Caspase-3 Activity Assay

The activity of caspase-3 was measured using a caspase-3 assay kit (fluorometric) according to the manufacturer’s instructions. Briefly, equal cells (1 × 10^6^ cells) of each treatment, including floating and adherent cells were collected, washed with PBS, and incubated in 60 μL chilled cell lysis buffer on ice for 10 min. After incubation, 50 μL lysates were transferred into a 96-well plate. Fifty microliters 2×reaction buffer and 5 μL DEVD-AFC (Asp-Glu-Val-Asp-7-amino-4-trifluoromethylcoumarin) substrate were then added into each sample in the 96-well plate for another 2 h incubation at 37 °C. Cleavage of the AFC peptides by caspase-3 releases the fluorochrome (excitation/emission = 400/505). The fluorescence signal was detected with a fluorescent microplate reader. Caspase-3 activity was presented as the percentage of the control.

### 4.6. Hoechst 33342 Staining

Hoechst 33342 (a DNA-specific fluorescent dye) staining was carried out to detect the alteration of the nuclear morphology of Hela cells after luteoloside treatment. In brief, the Hela cells in 1 mL media were seeded in 24-well plates. Following overnight adherence, the media were exchanged with fresh media containing various doses of luteoloside (0, 6.25, 25, and 100 μM). After incubation for the indicated time, Hoechst 33342 was added to the treated cells at a final concentration of 10 μg/mL, followed by incubation for another 10 min at 37 °C. The stained cells were then observed and photographed to examine the nuclear morphology under a fluorescence microscope equipped with standard excitation filters (Nikon, Tokyo, Japan).

### 4.7. DNA Fragmentation Assay

DNA of luteoloside-treated cells (1 × 10^6^ cells) was extracted using a DNA ladder extraction kit following the manufacturer’s instructions. The obtained DNA samples (approximately 20 μg) were electrophoresed at 50 V for 90 min in 1.5% agarose gel containing 1/10,000 Gel-Red (ethidium bromide substitute). The gels were then visualized and photographed under ultraviolet light.

### 4.8. ROS Levels Assay

ROS levels were measured with fluorescence probe DCFH-DA. DCFH-DA will be hydrolyzed to non-fluorescent 2′,7′-dichlorodihydrofluorescein (DCFH) in cells, which can be oxidized to fluorescent 2′,7′-dichlorofluorescein (DCF) by ROS. In brief, Hela cells were seeded in 96-well plates. Following overnight adherence, the media were exchanged with fresh media containing various doses of luteoloside (0, 6.25, 25, and 100 μM). After incubation for 8, 24 and 48 h, the media were removed and fresh media containing 10 μM DCFH-DA were added into it for another 20 min incubation at 37 °C. The cells were then washed again with fresh media three times, and the fluorescence intensity was analyzed on a microplate reader (BioTek Synergy2, BioTek Instruments Inc., Winooski, VT, USA) at 485/528 nm (excitation/emission).

### 4.9. Western BlotAnalysis

Cells were treated with luteoloside at the indicated concentrations and for different times, and then harvested. Subsequently, the cells were collected, washed twice with ice-cold PBS, and lysed in ice-cold RIPA buffer (containing protease and phosphatase inhibitor cocktail added just before use) for 10 min on ice. Cellular lysates were then centrifuged at 12,000 rpm for 10 min at 4 °C and the supernatants were collected. The protein content of the supernatants was determined with a BCA protein assay kit. Aliquots of protein sample (30 μg) were fractionated on 12% sodium dodecyl sulfate (SDS)-polyacrylamide gel and blotted onto PVDF membranes (Millipore). After being blocked with 5% (*w*/*v*) non-fat dry milk in PBS for 1 h at room temperature, the membranes were probed with the specific primary antibodies overnight at 4 °C. The membranes were then washed three times and exposed to horseradish peroxidase-conjugated secondary antibody for 2 h at room temperature. After the membranes were washed three times again, the antigen-antibody bands were visualized with an enhanced chemiluminescence (ECL) detection system. For each immunoblot, the experiments were repeated three times at least.

### 4.10. Statistical Analysis

Figures in the text are representative of at least three independent experiments. The data of the mitochondrial membrane potential correspond to the typical results presented. Other data are expressed as mean ± SD. Significant differences between the treated and untreated (control) cells were evaluated by the Student’s *t* test and indicated as * *p* < 0.05, ** *p* < 0.01, and *** *p* < 0.001. IC_50_ values of luteoloside on Hela, HUVEC12 and LO2 cells were calculated by regression analysis.

## 5. Conclusions

**Figure 9:**
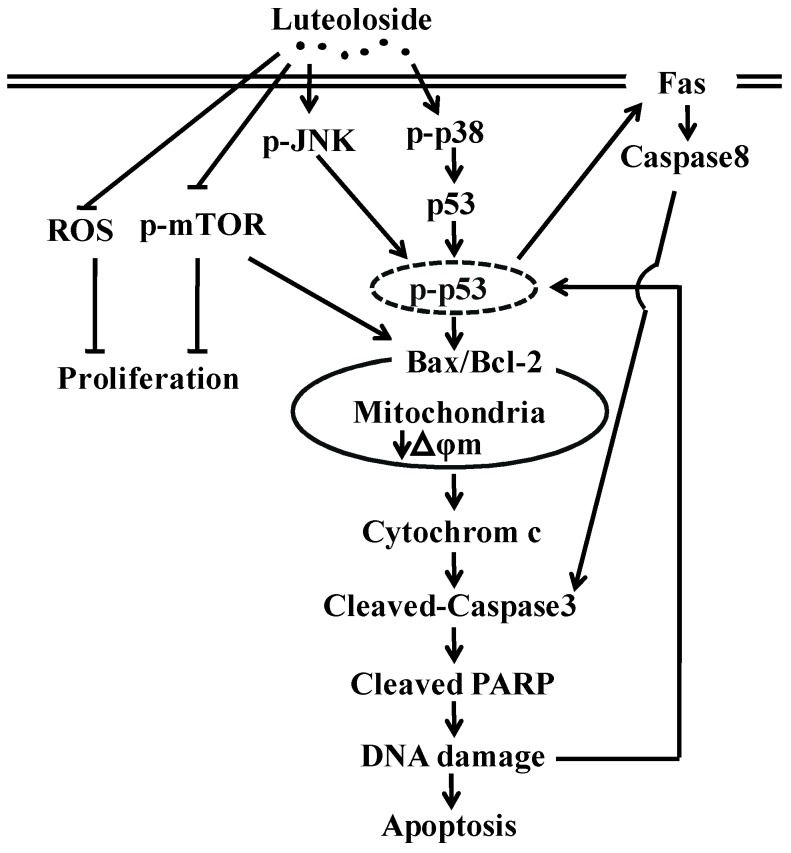
The possible signaling pathways for luteoloside-induced proliferation inhibition and apoptosis in Hela cells. ROS: Reactive oxygen species; PARP: Poly (ADP-ribose) polymerase; “⊥”: Inhibitory effect; “↓”: Stimulatory effect.

Figure 9 depicts the effects of luteoloside on Hela cells and the speculated mechanism. Luteoloside can inhibit proliferation and induce intrinsic and extrinsic pathway-mediated apoptosis in Hela cells. Furthermore, the effects of luteoloside may be achieved through inhibiting the mTOR signaling pathway and activating of the JNK and p38 MAPK two signaling pathways. Additionally, p53 is possibly involved in mediating the regulatory role of the JNK and p38 MAPK signaling pathways. In addition, luteoloside can reduce the ROS level of Hela cells, which may be responsible for the proliferation inhibition effect of luteoloside via the NLRP3 inflammasome. However, as an in vitro study, it would be very difficult to extrapolate the findings to the in vivo situation as is the case with many in vitro studies using cancer lines. Despite this, these findings provide support for the further investigation of luteoloside as a therapeutic agent for cervical cancer.
